# The relationship between acculturation strategies and depressive and anxiety disorders in Turkish migrants in the Netherlands

**DOI:** 10.1186/s12888-014-0252-5

**Published:** 2014-09-05

**Authors:** Burçin Ünlü Ince, Thijs Fassaert, Matty AS de Wit, Pim Cuijpers, Jan Smit, Jeroen Ruwaard, Heleen Riper

**Affiliations:** Department of Clinical Psychology, VU University Amsterdam, Van der Boechorststraat 1, 1081 BT Amsterdam, The Netherlands; EMGO Institute for Health and Care Research (EMGO+), VU University Medical Center, Amsterdam, The Netherlands; Public Health Service Amsterdam, Epidemiology and Health Promotion, Amsterdam, The Netherlands; Division of Online Health Training, Innovation Incubator, Leuphana University, Lueneburg, Germany; GGZ inGeest, Regional Mental Health Service Centre, VU University Medical Centre, Amsterdam, The Netherlands

**Keywords:** Acculturation, Integration, Assimilation, Separation, Marginalization, Depression, Anxiety, Migrants

## Abstract

**Background:**

Turkish migrants in the Netherlands have a high prevalence of depressive and/or anxiety disorders. Acculturation has been shown to be related to higher levels of psychological distress, although it is not clear whether this also holds for depressive and anxiety disorders in Turkish migrants. This study aims to clarify the relationship between acculturation strategies (integration, assimilation, separation and marginalization) and the prevalence of depressive and anxiety disorders as well as utilisation of GP care among Turkish migrants.

**Methods:**

Existing data from an epidemiological study conducted among Dutch, Turkish and Moroccan inhabitants of Amsterdam were re-examined. Four scales of acculturation strategies were created in combination with the bi-dimensional approach of acculturation by factor analysis. The Lowlands Acculturation Scale and the Composite International Diagnostic Interview were used to assess acculturation and mood and anxiety disorders. Socio-demographic variables, depressive, anxiety and co-morbidity of both disorders and the use of health care services were associated with the four acculturation strategies by means of Chi-Squared and Likelihood tests. Three two-step logistic regression analyses were performed to control for possible, confounding variables.

**Results:**

The sample consisted of 210 Turkish migrants. Significant associations were found between the acculturation strategies and age (*p* < .01), education (*p* < .01), daily occupation (*p* < .01) and having a long-term relationship (*p* = .03). A significant association was found between acculturation strategies and depressive disorders (*p* = .049): integration was associated with a lower risk of depression, separation with a higher risk. Using the axis separately, participation in Dutch society showed a significant relationship with a decreased risk of depressive, anxiety and co-morbidity of both disorders (OR = .15; 95% *CI*: .024 - .98). Non-participation showed no significant association. No association was found between the acculturation strategies and uptake of GP care.

**Conclusions:**

Turkish migrants who integrate may have a lower risk of developing a depressive disorder. Participation in Dutch culture is associated with a decreased risk of depressive, anxiety and co-morbidity of both disorders. Further research should focus on the assessment of acculturation in the detection of depression.

## Background

Research shows that the prevalence of depressive symptoms is significantly higher among adult ethnic minorities than among native populations in Europe [1]. In the Netherlands, it was found that Turkish migrants, one of the largest ethnic minorities in the Netherlands, have a significantly higher 1-month prevalence of depressive and/or anxiety disorders (18.7%) in comparison with Dutch (6.6%) as well as other ethnic minorities such as Moroccans (9.8%) [2]. Moreover, Turkish women especially, have a higher risk of prevalence of these affective disorders. However, in an international comparison study, it was shown that the 1-month prevalence of depressive and anxiety disorders for Turkish nationals living in Turkey was 3.1% [3]. It seems thus that the higher prevalence of depression among Turkish migrants may be related to migration.

While the Turkish population is at an increased higher risk for developing depression in comparison to other ethnic groups (Moroccan and Dutch), this is comparable with research concerning ethnic minorities in general. For example, in a European study in 23 countries, it was found that depressive symptoms were more prevalent among immigrants and ethnic minorities than among native populations [1]. A possible explanation for this higher risk may be lower socio-economic status and discrimination perceived by ethnic minorities in their host countries [1]. This increased risk for developing depression, therefore, is probably generalizable to many ethnic minority groups, including Turkish migrants in the Netherlands.

One of the first definitions of acculturation was given by Redfield and colleagues [4] in 1936 as “the changes that occur in the ethnic cultural patterns when groups or individuals with different cultures come into continuous contact with each other” (page 149)”. Later in 1980, Berry [5] applied this definition as a basis for his bi-dimensional model in which he defines acculturation as a) the degree of participation in the host culture by migrants and b) the degree of maintenance of their own ethnic culture. These two dimensions can vary independently of each other and can lead to four acculturation strategies according to Berry [6]. The first strategy can be described as *integration*, the combination of maintenance of the ethnic culture and participation in the host culture. *Assimilation* is the second strategy, which consists of participation in the host culture, but rejection of the original ethnic culture. Third, *separation (*or *segregation)* implies maintenance of the ethnic culture, but no participation in the host culture; and finally *marginalization,* when both the host and ethnic cultures are rejected.

In the past decades, alternative definitions have been given to acculturation, such as a second-culture acquisition [7] (p. 106) or enculturation [8] (p. 125). Both of these conceptualizations are viewing acculturation uni-dimensional, as one particular culture [9]. When viewing acculturation in a one-dimensional manner, the migrant chooses either to adapt the host culture or to maintain the ethnic culture. However, this one-dimensional approach neglects the dynamic of acculturation. According to the very first definitions of acculturation by Redfield, acculturation includes the interplay or transmission of one or more cultures, which is a criterion for acculturation nowadays [9]. The bi-dimensional model posits the independency of the two cultural orientations, which is shown to be a more valid approach of acculturation [10]. Therefore, its assessment by defining it in acculturation strategies has become an essential feautre of acculturation (e.g. [9,11,12]).

The integration strategy has often been associated with better psychological outcomes in comparison with the other three acculturation strategies. For example, migrants who are better integrated in the host culture show higher self-esteem, more prosocial behaviours and less depressive symptoms (e.g. [13,14]). In a recent meta-analysis by Gupta and colleagues [15] based on 38 studies on Asian Americans, it was found that participation in the American culture was related to lower depression scores among Asian Americans. Furthermore, they found that maintenance of the Asian culture had a negative but non-significant relationship with depression scores. Although acculturation was measured according to a bi-dimensional model (participation in the host country or maintenance of the ethnic culture), the combination of both strategies was not analysed by Gupta and colleagues [15].

Most research on ethnic minorities and mental health shows a negative association between acculturation and mental health. For example, in a study among Korean immigrants in the USA, self-reported language profiency of English (which is part of the adaptation dimension) was shown to be related with depression [16]. Furthermore, integration has been shown to be associated with lower mental health problems in Black male adolescents in the UK [17]. In Chinese American students, it was found that maintenance of the ethnic (Chinese) culture was related to fewer depressive symptoms [18].

However, there are also examples of studies in which this association hasn’t been found. For example, Beirens and Fontaine (2010) [19] evaluated differences in well-being in Turkish immigrants in Belgium, Turkish majority members (in Turkey) and Belgian majority members. Results showed no relationships were found between adaptation and maintenance (which were the only two acculturation dimensions) and sadness, anxiety nor with anger.

In the Netherlands, it was found that having fewer skills to enable participation in the host culture is generally related to more psychological distress [20–22]. This association was also confirmed for Turkish migrants, for example, by Fassaert and colleagues (2011) [23]. However, previous Dutch studies have not analysed acculturation according to the aforementioned four acculturation strategies (e.g. [20–25]). It is not yet clear whether the acculturation strategies are associated with affective disorders in Turkish migrants in the Netherlands. Since the literature shows that an integration strategy is related to lower levels of psychological distress in migrants, it is important to evaluate this explicitly in order to improve our understanding of the relationship between acculturation strategies and the prevalence of affective disorders among migrants.

In addition, ethnic minorities seem generally to receive less help from mental health care services than native citizens of Western countries [e.g. [26–29]). One of the reasons for this lower uptake is that ethnic minorities seek mental health care often at a later and at a more advanced stages of their mental health problems [16,17]. Moreover, ethnic minorities have a higher chance of dropping out from psychological treatment prematurely [18]. Although Dutch national data is lacking, there are signals that the dropout rate is twice as high in ethnic minorities in mental health care compared to native Dutch people in the Netherlands [30]. Research also shows that the perceived need for mental health care is higher for Turkish migrants than in Moroccan and Dutch people [24]. This may be related to their higher levels of mental distress and their less often met need perceived by Turkish migrants [24].

Several studies suggest that greater participation in the host culture is associated with higher general health service use (e.g. [31,32]). Earlier Dutch research found a significantly positive association between communication in Dutch and the use of care from General Practitioners (GPs) among Turkish men in the Netherlands [25]. However, an association between the four acculturation strategies and GP-care uptake by migrants was also not studied in the study of Fassaert and colleagues (2009) [25].

We therefore decided to examine the relationship between the four acculturation strategies (integration, assimilation, separation and marginalization) and mental health in terms of prevalence of depression and anxiety disorders in Turkish migrants living in Amsterdam as well as the association between these strategies and GP-care uptake. For this purpose we re-examined existing data from the General Health Monitor of Amsterdam of 2005 (e.g. [33]). Using the bi-dimensional framework of acculturation, we hypothesized that 1) higher integration is associated with lower prevalences of depression and anxiety disorders and 2) higher integration is associated with higher GP-care uptake.

## Methods

### The Amsterdam health monitor

The Amsterdam Health Monitor (AHM) consists of cross-sectional health surveys conducted by the public health service of Amsterdam (GGD). These surveys are performed periodically (every four years) in order to monitor the general health of the population living in Amsterdam and are representative for the population. Data for the current study were derived from the survey conducted in 2005, which was a (follow-up) study of the AHM of 2004 (GGD Amsterdam), stratified by age and ethnicity, and specifically aimed at studying mental health. The follow-up consisted of structured (diagnostic) interviews conducted by trained bilingual lay interviewers.

The first phase of the survey included 1449 respondents from the four largest ethnic minority groups (479 Dutch, 374 Moroccan, 454 Turkish and 142 Surinamese or Antillean). All of these respondents were asked for a second approach, without mentioning the topic of the study, one year after the first phase. This second phase (follow-up, one year after the first phase) consisted of a structured interview conducted by bilingual interviewers. A total of 1210 respondents gave permission to be approached for the follow-up study by invitation letter for a home visit by the interviewer. Appointments for a home visit could be changed by telephone, up to 8 attempts. The interviews took place between February and June 2005, while summer vacation, Christmas and Ramadan were avoided. The interviews could be held in Dutch, Turkish, Moroccan or Berber, depending on the preference of the respondent. The interviewers were intensively trained and coached before and during data-collection. The interviews were also recorded in order to check and coach the researchers. After completing the interviews, these were checked for consistency and completeness.

The second phase resulted in 812 respondents, of whom 321 were Dutch, 191 Moroccan, 213 Turkish and 87 Surinamese or Antillean. All the study procedures were approved by the ethics commission of the Amsterdam Academic Medical Center. For more detailed information about the recruitment, procedures and details, we refer to previous AHM publications (e.g. [2,23,24]). For the current study, we selected the data of the Turkish group, comprising 210 respondents with complete information (Table 1).

**Table 1:** **Characteristics of the Turkish group from the AHM of 2005**

	**Turkish migrants**	
	**N = 210**	
	n	%
Gender		
Male	126	60.0
Female	84	40.0
Age (M,SD)	47.4	14.2
18 - 35	44	21.0
36 - 49	74	35.2
≥ 50	92	43.8
Education level^a^		
None or primary school	103	49.0
Middle education.	35	16.7
Higher education	48	22.9
Daily occupation		
Yes, job/student	60	28.6
No, unemployed.	150	81.4
Partnership^b^		
Yes, partner	169	80.5
No, single	38	18.1
Acculturation^c^		
Integration	41	19.5
Assimilation	44	21.0
Separation	87	41.4
Marginalization	24	11.4
Depression and dysthymia (1-month)	36	17.1
Anxiety disorders (1-month)	21	10.0
Comorbidity	14	6.7
Amount of the contacts with GP^d^		
No contact (0)	58	27.6
Low (0–3)	90	42.9
High (>3)	61	29.0

*Note.* Results are based on the completers only sample due to varying attrition rates on measurements. Numbers do not add up to 210, because not all participants answered all the questions. Missing values are noted in superscript: a: *n* = 24; b: *n* = 3; c: *n* = 14 and d: *n* = 59.

### Measures

The structured diagnostic interview consisted of several translated instruments, of which we selected only the following sections: demographic information, the Lowlands Acculturation Scale and the Composite International Diagnostic Interview and the use of health care services. The questionnaire was translated into Turkish by official translators. A back translation to Dutch was performed by another translator and checked by the researchers. Any inconsistencies with the original were discussed with both translators and adjusted. The interviewers also reported back when they had difficulties with the translation, and then together a standard was chosen. Of the acculturation scale (LAS) and the measurement of anxiety and depression (CIDI) official Turkish translations were available and used in this study.

### Socio-demographic variables

Socio-demographic information included gender, age (18–35 years, 36–49 and 50 years and older), level of education (none, to at most 6 years of primary schooling; up to 10 years of schooling including middle education; up to 18 years of schooling including higher education), daily occupation (employed for more than 12 hours a week; a student; or no job) and partnership status (having a long-term relationship or partner). Turkish ethnicity was defined as when the respondent or at least one of his/her parents was born in Turkey. First and second generation migrants were taken together as one group. These items were translated from Dutch to Turkish and back to Dutch by professional translators.

### Acculturation

The level of acculturation was measured with the Lowlands Acculturation Scale (LAS), which was used in the AHM of 2005 [34]. The validated Turkish translation was used [34]. It consists of 25 items that are rated on 6-point Likert-type scales, ranging from ‘totally disagree’ to ‘totally agree’. The LAS can be divided into 5 subscales: Skills, Traditions, Social Integration, Values and Norms; and Feelings of Loss.

However, for the purpose of the research question addressed in this paper, we did not use the original scales of the LAS. We developed four new acculturation strategy scales based on the items of the LAS questionnaire (integration, assimilation, separation and marginalization) in two steps:

First, following the two-dimensionality theory of Berry [5,6], two new scales were developed for the questionnaire. These scales were: participation and contact in the host culture (Participation) and maintenance of the ethnic culture (Maintenance). The two-dimensionality of the items on the LAS questionnaire was created by explorative factor analysis (principal component analysis) with a two-factor solution, based on the respondents of the AHM 2005. However, eight items concerning emancipation were excluded from further analyses because it was not possible to determine how this scale was associated with acculturation, due to the lack of information about emancipation in the ethnic Dutch and Turkish cultures. The two factors, participation and maintenance, were yielded with the rotation solution, as shown in Table 2. The participation factor accounted for 16.9% of the item variance, and the maintenance factor accounted for 21.6% of the item variance. With a cut-off point of .40 for the loadings [35], only 3 items were excluded from both factors. In order to fit the LAS items to the scales, some items (items 10, 11, 13, 16 and 17) were recoded by adjusting the range of the response options, so that higher scores indicated lower levels of maintenance or participation. Internal consistency for the Participation and Maintenance scales of the LAS were good, with Cronbach’s alpha indicating strong reliability for both of the scales - each .86.

**Table 2:** **Factor loadings of the items on the LAS**

	**Factors**	
**Items**	**Participation**	**Maintenance**
*Participation items*		
11. I find Dutch difficult, so I’m not motivated to learn	**.67**	.30
15. I am misunderstood when I speak Dutch	**.70**	.26
16. I have difficulties understanding the Dutch language	**.80**	.36
17. I have to depend on other people to show me how things are done here	**.77**	.22
20. I must learn how certain tasks are done, such as renting an apartment	**.51**	.25
*Maintenance items*		
2. I prefer to listen to Turkish music	.15	**.53**
4. I prefer to eat Turkish food	.17	**.59**
6. I consider it important to pass our traditions on to the next (future) generation	.04	**.61**
8. It is important to me to celebrate the Turkish traditional feast in the Netherlands	.11	**.56**
10. I belong here less than I belong to my homeland	.24	**.50**
12. When I go out, I usually go to places where I can meet people from my home country	.33	**.52**
13. Even though I am living here, it does not feel like my country	.32	**.44**
14. Most of my friends have the same cultural background as I do	.32	**.60**
22. My country of origin is always on my mind and in my memories	.11	**.64**
24. I miss the people I left behind in my original country	.21	**.67**
25. I feel homesick	.26	**.65**
*No Factor items*		
5. I have frequent contact with Dutch people	.26	-.19
7. In my experience encounters with the Dutch are fine	-.17	.07
18. I am familiar with the Dutch politics	-.29	-.11

*Note.* Factor loadings above.40 are presented in bold.

Second, in order to combine participation (low or high) and maintenance (low or high) to create the 4 acculturation strategies, the medians of the two scales were used as the cut-off scores indicating high (higher or equal to the median) and low (lower than the median) levels of participation or maintenance. Choosing a median split instead of a continuous measure was based on the fact that the distribution of the dimensions was not normal distributed, and therefore the dimensions could not be validly included as continuous measures and were therefore dichotomized. This also made it feasible to compare the four strategies instead of two separate dimensions. The median for participation was 20.0 (range: 5–30) and for maintenance it was 50.0 (range: 25–66).

This resulted in four scales of acculturation strategies: the scale *integration* was composed of the combination of high participation and high maintenance. The scale *assimilation* was composed of the combination of high participation and low maintenance. The scale *separation* consisted of low participation and high maintenance. Finally, the scale marginalization was the combination of lower levels of both participation and maintenance.

### Anxiety and depressive disorders

The Composite International Diagnostic Interview (CIDI 2.1) was used to establish the presence of depressive and anxiety disorders [36]. Depressive disorders included major depressive disorder and dysthymia; anxiety disorders included social phobia, agoraphobia, panic disorders and generalised anxiety disorders. All disorders were coded according to the DSM-IV criteria [37]. The WHO Turkish version of the CIDI was used and was conducted by trained lay interviewers.

### Use of health care services

The outcome measure for health services utilisation was evaluated in terms of contacts with a General Practitioner (GP) by a self-report measure. Contacts were defined as consulting hours, telephone contacts, number of consultations with the GP for general health in the 6 months preceding the interview. A distinction was made between low and high number of contacts (0 to 3 versus more than 3 contacts).

### Analyses

Socio-demographic variables, depressive disorder, anxiety disorders, co-morbidity and the use of health care services were analysed in terms of associations with acculturation strategies. To assess the significance of an association between these variables and the acculturation strategies, Pearson’s Chi-Squared test was used. For cross tabulations with low cell frequencies (<5) the Likelihood ratio test was performed Acculturation was also analysed in terms of a division in two general dimension (participation in the Dutch society and non-participation in the Dutch society) in order to explore one specific cultural attitude. To control for possible confounding variables, we conducted three two-step logistic regression analyses, with the binary (yes/no) psychopathology variables (depression/dysthymia, anxiety and comorbidity) as dependent variables. In step 1 of the analysis, we entered the five socio-demographic variables listed as an independent variable (Model 1). In step 2, we added the four acculturation variables (Model 2). Next, a Chi-Squared test of the log likelihood of model 1 versus model 2 was used to test the relationship between acculturation and psychopathology, taking into account the effects of socio-demographic variables. Associations were considered statistically significant if *p* < .05. All analyses were conducted in SPSS 20.0.

## Results

### Socio demographic characteristics of participants

The sample consisted of 210 Turkish migrants, as shown in Table 1. More than half of the migrants were male (60%) and most were over 36 years of age (79%). Almost half of the participants had only primary education and 80% were unemployed. Likewise, 80% were in a long-term partnership.

The 1-month prevalence of depression and dysthymic disorders was 17.1%, while the 1-month prevalence of anxiety disorders was 10%. The prevalence of co-morbidity of both disorders was high, namely 6.7%. Almost half of the of the participants (42.9%) reported 0 to 3 contacts with their GP for their general health in the last 6 months, of whom 64.4% (n = 58) had no contact at all. Finally, 29% had contact with their GP on more than 3 occasions.

### Acculturation and demographic characteristics

Several associations were found between acculturation strategies and demographic characteristics, as shown in Table 3. Results show that age (Likelihood ratio = 40.79, *p* < .001), education (Likelihood ratio = 59.51, *p* < .001), daily occupation (*χ*^2^ (4) = 32.22, *p* < .001) and partnership status (*χ*^2^ (3) = 9.12, *p* = .03) were significantly associated with acculturation. Gender did not show an association with acculturation (***χ***^**2**^ (3) = 6.20, *p* = .10).

**Table 3:** **Associations between acculturation strategies and demographic characteristics, mood/anxiety disorders and contact with GP (** ***n*** **,%)**

	**Acculturation strategies**				**Statistics**	
	**Integration**	**Assimilation**	**Separation**	**Marginalization**	**Chi-Squared**	**df**
	n = 41	n = 44	n = 87	n = 24		
Demographic characteristics						
Age					40.79ª ***	6
18-35	14 (34.1%)	20 (45.5%)	8 (9.2%)	0 (0%)		
36-49	13 (31.7%)	15 (34.1%)	32 (36.8%)	10 (41.7%)		
≥ 50	14 (34.1%)	9 (20.5%)	47 (54.0%)	14 (58.3%)		
Gender					6.20	3
Female	26 (63.4%)	31 (70.5%)	48 (55.2%)	10 (41.7%)		
Male	15 (36.6%)	13 (29.5%)	39 (44.8%)	14 (58.3%)		
Education^b^					59.51ª ***	6
low	15 (36.6%)	8 (18.2%)	61 (70.1%)	13 (54.2%)		
middle	7 (17.1%)	15 (34.1%)	8 (9.2%)	2 (8.3%)		
high	16 (39.0%)	19 (43.2%)	4 (4.6%)	7 (29.2%)		
Daily pursuits^c^					32.22***	3
Job/student	14 (34.1%)	26 (59.1%)	11 (12.6%)	5 (20.8%)		
Unemployed	27 (65.9%)	18 (40.9%)	76 (87.4%)	19 (79.2%)		
Partnership^d^					9.12*	3
Yes, partner	34 (82.9%)	32 (72.7%)	75 (86.2%)	16 (66.7%)		
No, single	7 (17.1%)	12 (27.3%)	9 (10.3%)	8 (33.3%)		
Mood/anxiety disorders						
Depression/Dysthymia						
Yes	2 (4.9%)	8 (18.2%)	20 (23.0%)	5 (20.8%)	7.85*	3
No	39 (95.1%)	36 (81.8%)	67 (77.0%)	19 (79.2%)		
Anxiety disorders						
Yes	2 (4.9%)	2 (4.5%)	12 (13.8%)	5 (20.8%)	6.85	3
No	39 (95.1%)	42 (95.5%)	75 (86.2%)	19 (79.2%)		
Co-morbidity						
Yes	1 (2.4%)	1 (2.3%)	10 (11.5%)	2 (8.3%)	6.08	3
No	40 (97.6%)	43 (97.7%)	77 (88.5%)	22 (91.7%)		
Contact with GP						
Frequency of contacts^e^					3.73	3
Low (0–3 )	16 (39.0%)	23 (52.3%)	35 (40.2%)	11 (45.8%)		
High (>3)	9 (22.0%)	9 (20.5%)	32 (36.8%)	8 (33.3%)		

Note. a = The Likelihood Ratio Value is provided due to low cell frequency (<5). Results are shown based on the completers only sample due to varying attrition rates on measurements. Numbers do not add up to 210, because not all participants answered all the questions. Missing values are noted in superscript: b: *n* = 35; c: *n* = 14; d: *n* = 17 and e: *n* = 67.

* = *p* < .05; ** = *p* < .01; *** = *p* < .000.

Table 3 also illustrates the proportions of demographic characteristics of migrants in the four acculturation strategies. The acculturation scale separation had the highest percentage of migrants (41,4%, n = 87) and marginalization had the lowest percentage of migrants (11.4%, n = 24).

The integration scale consisted mainly of migrants who were aged between 18 and 35 (34.1%, *n* = 14) and 50 years or older (34.1%, *n* = 14) and unemployed (65.9%, *n* = 27). The assimilation scale included migrants with slightly different characteristics than for the integration scale, namely those aged between 18 and 35 (45.5%, *n* = 20), were employed or were students (59.1%, *n* = 26).

The separation scale comprised migrants with a different profile than the previous scales, i.e. 54.0% (*n* = 47) were older migrants, 55.2% (*n* = 48) were female, 70.1% (*n* = 61) had a lower educational level and 87.4% (*n* = 76) were unemployed. The marginalization scale had a similar profile of migrants to those in the separation scale, with the exception of the relationship status. In the separation scale 33.3% of the migrants (*n* = 8) had no relationship.

### Acculturation and depressive/anxiety disorders

Table 3 also presents the results of the association between acculturation and depressive and anxiety disorders. Acculturation was significantly associated with depressive disorders (Likelihood ratio = 7.85, *p* = .049), but not with anxiety disorders (Likelihood ratio = 6.85, *p* = .08) nor with co-morbidity of these disorders (Likelihood ratio = 6.08, *p* = .11).

Migrants who had a depression diagnosis (*n* = 36) were mainly represented in the separation scale (*n* = 20), while the integration scale had the lowest number of migrants with depression (*n* = 2).

### Acculturation and anxiety/depressive disorders controlled for socio-demography

Tables 4 and 5 present the results of the three regression analyses we conducted in order to control for possible cofounders. Results were similar to the results of the original analyses presented in Table 6. Accounting for the effects of socio-demographic variables, acculturation strategies were related to depression, but not to anxiety although the relationship with anxiety approached significance (*p* = .055). Risk of depression was significantly lower among those adopting an integration strategy (OR = .15; 95% CI: .024- .98).

**Table 4:** **The association between Acculturation and 1-month prevalence of mood/anxiety disorders, controlling for socio-demographic variables**

	**−2 Log Likelihood**		**Chi-Square**		
	**Model 1**	**Model 2**	**Statistic**	**df**	**P-Value**
Depression/Dysthymia	153.05	145.18	7.87	3	.049
Anxiety disorders	109.67	102.08	7.59	3	.055
Co-morbidity	82.86	76.81	6.05	3	.11

*Note.* Results are based on the completers-only sample. Model 1: includes the five socio-demographic variables as an independent variable. Model 2: acculturation was added as a variable.

**Table 5:** **The association between integration/assimilation and 1-month prevalence of mood/anxiety disorders, controlling for socio-demographic variables**

	**−2 Log Likelihood**		**Chi-Square**		
	**Model 1**	**Model 2**	**Statistic**	**df**	**P-Value**
Depression/Dysthymia	153.05	147.95	5.10	1	.02
Anxiety disorders	109.67	102.09	7.58	1	.01
Co-morbidity	82.86	77.33	5.52	1	.02

*Note.* Results are based on the completers-only sample. Model 1: includes the five socio-demographic variables as an independent variable. Model 2: acculturation was added as a variable.

**Table 6:** **Acculturation strategies in two categories and 1-month prevalence of mood/anxiety disorders (** ***n*** **,%)**

	**Acculturation strategies**		**Statistics**	
	**Participation**	**Non-participation**	**Chi-Squared**	**df**
	n = 85	n = 111		
Depression/Dysthymia			3.80	1
Yes	10 (11.8%)	25 (22.5%)		
No	75 (88.2%)	86 (77.5%)		
Anxiety disorders			4.61ª *	1
Yes	4 (4.7%)	17 (15.3%)		
No	81 (95.3%)	94 (84.7%)		
Co-morbidity			4.00ª *	1
Yes	2 (2.4%)	12 (14.1%)		
No	83 (97.6%)	99 (89.2%)		

*Note.* Results are based on the completers-only sample. a = The Likelihood Ratio Value is provided due to low cell frequency (<5); * = *p* < .05; ** = p < .01 and *** = *p* < .000.

### Combining acculturation strategies

Additional analyses were performed on the basis of a division into two combined acculturation strategies: (1) participation in the host culture consisting of integration and assimilation and (2) non-participation in the Dutch society consisting of separation and marginalization, shown in Table 6. The two combined strategy categories showed no significant association with depressive disorders, however there was a trend towards statistical significance (*χ*^2^ (1) = 3.80, *p* = .051).

For anxiety disorders there was a significant association between the two acculturation categories (Likelihood ratio = 4.61, *p* = .03). Finally, co-morbidity likewise showed a significant association between the two general acculturation strategies (Likelihood ratio = 4.00, *p* = .02). For all three measures of psychopathology results showed a higher prevalence among those with a non-participatory strategy.

Next, we focused on the same regression analysis method to examine the relationship between *acculturation strategies* (divided into two general categories*)* and *psychopathology* (comparing participation to non-participation). As shown in Table 5, the acculturation-strategy category of participation did relate to each of the three measures of psychopathology. Participation was associated with decreased risk of depression (OR = .30, 95% CI: .10 - .89), anxiety (OR = .13, 95% CI: .03- .66) and co-morbidity (OR = .12, 95% CI: .02 - .90).

### Acculturation and the use of health care services

As presented in Table 3, acculturation did not show an association with the frequency of contacting the General Practitioner (*χ*^2^ (3) = 3.73, *p* = .29).

## Discussion

In this paper, the relationship between four acculturation strategies (integration, assimilation, separation and marginalization) and depressive/anxiety disorders among Turkish migrants living in Amsterdam was examined. For this purpose we used existing data from the General Health Monitor of Amsterdam, dating from 2005 [e.g. [2,23–25,33].

Results showed that age, education, daily occupation and partnership status were significantly associated with acculturation. We also found a significant association between the acculturation strategies and depressive disorders. Migrants who adopted the integration strategy had a significantly lower risk of depression compared to those with one of the other three strategies. There was no association between any of the four acculturation strategies and the frequency of contacting the General Practitioner.

When the four acculturation strategies were combined into two categories (defined as either participation in the Dutch culture (integration and assimilation) or non-participation in the Dutch society (separation and marginalization), our results suggest that participation in the host country seems to be associated with lower risk of depressive and anxiety disorders and co-morbidity of both these disorders.

It is noteworthy to mention that the migrants included in the study have a high level of unemployment (over 80%) across all acculturation strategies. The unemployment rate in our sample was much higher than found in the general Turkish population in the Netherlands, which was 14.8% in 2005 [38]. This means that our results are mainly indicative for unemployed Turkish migrants. Furthermore, migrants who applied a participatory strategy (integration and assimilation) were often young, female and higher educated, while those in the non-participating strategy (separation and marginalization) comprised mainly migrants aged 50 or older, who were lower educated. Several studies have shown that a low socio-economic status (SES) is a risk factor for developing mental health disorders in general and also for developing depression (e.g. [39–41]. Although, after correction for the socio-economic variables the association were still significant, we could not examine whether the association might be different within different levels of socio-economic status (SES).

### Comparison with prior work

Our results are in line with the general finding that an integration strategy among migrants is associated with better psychological outcomes as found in the studies by Chen and colleagues [13] and Schwartz et al. [14]. These authors found that migrants who hold an integration strategy experience higher self-esteem and less depressive symptoms [13,14]. Moreover, the two combined acculturation strategies (participation in the host country and non-participation in the host country) seem to be independent mechanisms, as participation is associated with depression while non-participation is not, corresponding to the independent bi-dimensional theory of Berry [5,6].

There are number of possible explanations for the lower risk of depression among migrants with participatory acculturation strategies. Earlier research showed that migrants who have integrated into the host society, have cultural knowledge about the host society, are better able to control the degree of contact and have positive cultural group attitudes. All these factors may contribute to minimising cultural distance between migrants and their host society [42,43]. In turn, integration may enable migrants to manage their daily life in the host society better and therefore lower their risk of depression. Since integration involves a positive multicultural attitude [44] that enables migrants to manage daily life in a new context, it is likely that these behaviours play a role in a decreased prevalence of depression in Turkish migrants who adopt such a strategy.

Finally, our results showed no association with the acculturation strategies and uptake of GP care and thereby confirmed the generic analysis of acculturation and GP care uptake by Fassaert and colleagues [25]. However, our results are not in line with earlier research (e.g. [31,32]) which showed that participatory strategies were associated with higher use of general health care services. For example, having higher levels of skills to participate in Dutch society was related to greater use of health care by migrants in the Netherlands [32]. It thus seems that Turkish migrants make use of GP services to the same degree, regardless of their acculturation strategy.

### Limitations

This study has several limitations. First, the definitions of immigrants/migrants will undoubtedly have affected the results. First and second generation immigrants were taken as equal groups in the analysis, because the number of second generation migrants in the study was too small (*n* = 16) to study this group separately. Therefore, these results mainly represent first generation migrants. Yet, it is likely that each of these groups will experience the process of acculturation through different (path)ways, which were not monitored. Second, the cross-cultural validity of the four created acculturation strategy scales was not tested, although the new scales showed good reliability (e.g. Cronbach’s alpha was .86 for both scales). The small size of the groups distinguished by acculturation strategy resulted in low power to detect possible associations and differences. We found several associations, however, not all were significant. Furthermore, the absence of the cross-cultural validity of the CIDI is also an important limitation. Third, the theoretical conceptualisation of acculturation is complex. We adopted the bi-dimensional model of Berry [5,6], however, acculturation does not take place in a ‘vacuum’. Acculturation is a dynamic process that encompasses not only certain life domains, but also contextual, political, economic and social factors that require further exploration [45]. It was beyond the scope of our study to include all these factors in our analyses. Furthermore, the response rate over the first and second phases was 26%. It is not clear why the response was that low. However, despite efforts put into reaching and recruiting ethnic minorities, it seems that this low response rate is the highest possible response to be attained in ethnic minorities [2,46]. There may have been a selective response, which is a limitation. However, the response rate of the Turkish group was similar to the other ethnic minorities in the data (Moroccan, Antillean and Surinamese), suggesting that in case of selective response this was similar in all the ethnic minority groups. Finally, the cross-sectional design of the study restricts the causality of the associations.

### Implications and future research

The finding that integration may play an important role in a lower risk of developing depression is also of importance for public health policy makers, clinicians as well as for researchers. Supporting immigrants in the process of adjustment to the host society, while encouraging ethno-cultural maintenance at the same time, is an important task for the Dutch society as well as for ethnic minorities themselves. This process can be aided through several pathways, including educational and public health policies, such as implementing acculturation in prevention programs. Although integration has been found to be related to a lower risk of depression, its causality and implications for prevention and clinical practice should be examined in more detail for example the potentials of including it as component in screening or treatment strategies. Awareness by practitioners and professionals of the acculturation strategies of ethnic minorities should be promoted in order to optimize health services for mental health problems. The assessment of the acculturation strategies migrants adopt may be useful in identifying high risk profiles of migrants who are at increased risk for depression. From a public health perspective it may thus be advised to include types of acculturation strategies in screening procedures for depression. It is also of importance to examine what factors are affecting the relationship between integration and depressive disorders and whether these also hold for the employed Turkish migrant population. Moreover, future research should also explore the influence of socio-economic status on the relationship between acculturation and depression.

## Conclusion

Turkish migrants who participate in Dutch society, while at the same time maintaining their ethnic culture, may have a lower risk of developing a depressive disorder compared to those who adopt other acculturation strategies. Participation in Dutch culture is associated with a decreased risk of depression/dysthymia, anxiety and co-morbidity of both disorders. No association was found between the acculturation strategies and GP care. Future research should focus on the assessment of acculturation in the detection of depression.

## Abbreviations

GP: General practitioner
AHM: Amsterdam Health Monitor
GGD: Public Health Service
LAS: Lowlands acculturation scale
CIDI: Composite international diagnostic interview
